# Ticagrelor-Induced Angioedema: A Rare and Unexpected Phenomenon

**DOI:** 10.1155/2017/7612713

**Published:** 2017-12-17

**Authors:** Rajeev Seecheran, Valmiki Seecheran, Sangeeta Persad, Sasha Lalla, Naveen Anand Seecheran

**Affiliations:** ^1^University of the West Indies, St. Augustine, Trinidad and Tobago; ^2^North West Regional Health Authority, Mt. Hope, Trinidad and Tobago; ^3^Advanced Cardiovascular Institute, Port of Spain, Trinidad and Tobago

## Abstract

Angioedema can cause potentially life-threatening airway obstruction. This case report describes an exceedingly rare episode of ticagrelor-induced hypersensitivity reaction, manifesting as angioedema with periorbital and likely respiratory involvement. The heart team should be vigilant for this precarious condition which may require emergent airway management. Desensitization protocols and alternative regimens (e.g., clopidogrel, prasugrel, and addition of an adjunctive anticoagulant) should be considered when there is an absolute indication for antiplatelet therapy.

## 1. Introduction

The benefit of dual antiplatelet therapy (DAPT) following an acute coronary syndrome (ACS) was established by several pivotal trials [1–3]. DAPT with clopidogrel reduced the 1-year incidence of cardiovascular events by approximately 20% compared with aspirin monotherapy. Subsequently, more potent and consistent P2Y12 receptor inhibition with either prasugrel or ticagrelor was superior to clopidogrel in the respective TRITON [4] and PLATO [5] trials.

Clopidogrel has become a mainstay of treatment of patients with ACS to reduce ischemic complications after percutaneous coronary interventions. Prasugrel, with its more potent effect, is the immediate successor to clopidogrel. The newest member of the P2Y12 inhibitors is ticagrelor, which is not a thienopyridine and, however, demonstrates a superior pharmacological profile than clopidogrel. In contrast to clopidogrel and prasugrel, it does not require metabolic activation and binds reversibly to the P2Y12 receptor [6].

As the use of clopidogrel has proliferated, hypersensitivity reactions have been increasingly recognized [7]. Generally, allergic reactions are frequent with antiplatelet drugs with aspirin being the chief culprit, with a prevalence of 1.5%. Hypersensitivity reactions occur in 6% of patients with clopidogrel [8]. There are case reports which identify allergic reactions with ticagrelor; however, the exact prevalence cannot be ascertained [9].

ACS patients receiving DAPT should be closely monitored for adverse drug reactions (ADRs). While clinical trials provide valuable information about common ADRs, it is crucial that rarer events are reported into pharmacovigilance databases that can be accessed by healthcare providers and patients alike [10].

## 2. Case Report

A 69-year-old African gentleman with a medical history of coronary artery disease after percutaneous coronary intervention to his right coronary artery in 2014 for a non-ST-segment elevation myocardial infarction and prior contrast-media allergy presented to the emergency department with atypical angina.

Shortly after his arrival, he was seen by the emergency medicine physician who initiated an acute coronary syndrome antiplatelet regimen comprising both oral aspirin 325 mg and ticagrelor 180 mg. Vital signs revealed mild hypertension with a blood pressure of 147/94 mmHg, normal pulse rate of 74 beats per minute, and an oxygen saturation of 99% on pulse oximetry on room air. His physical examination was significant for left-sided chest wall tenderness only. A 12-lead electrocardiogram (EKG) revealed sinus rhythm with a 1st degree atrioventricular block and a left anterior fascicular block with prior inferior myocardial infarction. There were no acute ischemic changes. His cardiac biomarkers were normal (peak serum levels of creatine phosphokinase-MB fraction (CK-MB) and troponin T were 1.5 ng mL^−1^ (normal range, 1–5 ng mL^−1^) and 0.034 ng mL^−1^ (normal range, 0-0.1 ng mL^−1^), respectively) and did not reflect a myocardial infarction.

**Figure 1:**
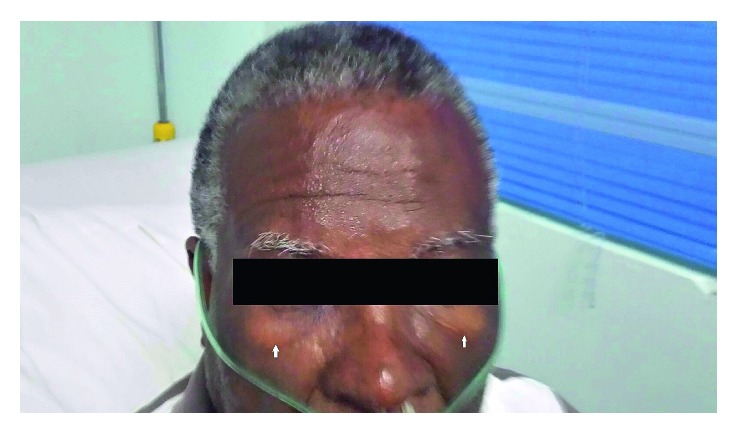
The white arrows indicate periorbital edema.

Approximately 1 hour after ticagrelor administration, the patient reported a constellation of symptoms which included worsening chest pain, sudden-onset respiratory distress, and generalized urticaria. His repeat physical examination revealed new-onset periorbital edema and angioedema (Figure 1). He was immediately administered 200 mg intravenous hydrocortisone, 100 mg intravenous ranitidine, and 10 mg intravenous chlorphenamine with nebulized albuterol and ipratropium, and his symptoms resolved shortly thereafter (within an hour).

Based on the clinical scenario, the patient's tentative diagnosis was ticagrelor-induced hypersensitivity reaction with angioedema as this was the only new medication he was given (he was on daily maintenance aspirin for his CAD and previously tolerated a 1-year course of clopidogrel after drug-eluting stent implantation). It was also noted that he was not on an angiotensin-converting enzyme inhibitor or angiotensin receptor blocker. He was subsequently transferred to cardiac care unit for further observation and supportive care. The following day, a 2D transthoracic echocardiogram revealed a preserved left ventricular ejection fraction of 60% without regional wall motion abnormalities. He reported that his symptoms were much improved and was hemodynamically stable. His clinical angioedema completely resolved. The patient's atypical angina completely abated, and subsequent cardiac biomarkers and electrocardiograms were unremarkable. As a result, inpatient coronary angiography was not pursued as his TIMI risk stratification was considered low to moderate risk, and outpatient exercise stress echocardiography was the preferred management strategy to assess for ischemia. He was safely discharged on guideline-recommended, optimal medical therapy comprising aspirin, clopidogrel (which he previously tolerated as DAPT for his prior stent implantation without any adverse effects), beta-blocker, mineralocorticoid receptor antagonist with high-intensity statin, and a tapered course of oral steroid therapy.

## 3. Discussion

Acquired angioedema (AAE) can be immunologic, nonimmunologic, or idiopathic [11]. It is generally characterized by repetitive episodes of swelling, and if it occurs in the upper respiratory tract, it can be imminently life-threatening [12, 13]. The pathophysiology is ascribed to the accumulation of bradykinin [13]. Angiotensin enzyme inhibitors and angiotensin receptor blockers are common agents which can elicit this condition; however, our patient's medical regimen did not comprise either. The estimated incidence of drug-induced angioedema is reported to be less than 1% [14].

In this case, ticagrelor, a cyclopentyl-triazolopyrimidine with a similar structure to adenosine, was administered in combination with aspirin. Periorbital edema was evident within an hour of administration and, thus, appeared to be temporally linked (Figure 1). Laryngeal and respiratory involvement were immediately considered with the onset of the patient's dyspnea. Histamine antagonists (both H_1_ and H_2_) and intravenous steroids are the mainstays of treatment, and in severe cases (e.g., airway obstruction or anaphylaxis), epinephrine may be warranted [14]. The goals of emergency treatment of angioedema are to prevent spontaneous eruption, to maintain a patent airway if eruption does occur, and to stop progression of disease [11]. As laryngeal edema progresses rapidly, stridor of the airway occurs with resultant hypoxia. Tracheal intubation is required in these situations to prevent respiratory arrest and risk of death. In these cases, a definitive airway such as an endotracheal tube should be established. If the airway cannot be effectively secured with an endotracheal tube, a surgical airway is indicated, usually in the form of an emergency cricothyrotomy [12].

Another important aspect to consider is that the patient had a previous allergic reaction to contrast media with urticaria during his prior percutaneous coronary intervention. In general, patients with unrelated allergies are at a 2- to 3-fold increased risk of an allergic-like contrast reaction, and this suggests that ticagrelor was the likely culprit for precipitating the angioedema given his background [15].

While ticagrelor is a recognized cause of angioedema, the literature is not replete with case reports or series describing the role of ticagrelor in angioedema. Clopidogrel, an oral thienopyridine prodrug, is generally well tolerated, but 1.5% of patients eventually require drug discontinuation [16]. Prasugrel, also a thienopyridine prodrug, is known to have caused similar immediate-type allergic reactions including angioedema, but the prevalence appears to be less frequent [17]. There are few reported cases of cross-reactivity between prasugrel and ticagrelor; however, the presence of cross-reactivity between clopidogrel and prasugrel appears to not be infrequent [18].

With respect to the clinical scenario warranting dual antiplatelet therapy for a protracted period, several therapeutic options can be considered. Cheema et al. [16] investigated patients with suspected hypersensitivity reactions to clopidogrel manifesting with facial angioedema, and the vast majority was able to continue treatment and experienced a resolution of symptoms under short-term systemic corticosteroids [19]. Another strategy is drug desensitization, which has proved to be successful in several specific cases and, in small case series, can be attempted in complex cases where there is an absolute indication for a P2Y12 inhibitor [8]. The conventional approach for a persistent reaction has been to substitute with alternative therapies [16, 19]. He was not subsequently challenged with either clopidogrel (which he previously tolerated without issue) or prasugrel, as he did not have a definitive myocardial infarction.

## 4. Conclusion

In summary, we describe a case report of ticagrelor-induced angioedema occurring within 1 hour after administration. The heart team should be vigilant for angioedema as an adverse drug reaction which can precipitate respiratory distress requiring emergent airway management. Desensitization protocols and alternative regimens (e.g., clopidogrel, prasugrel, and addition of an adjunctive anticoagulant) should be considered when there is the absolute indication for antiplatelet therapy.

## Additional Points

Ticagrelor can abruptly induce angioedema which may cause life-threatening airway obstruction requiring emergent airway management. Desensitization protocols and alternative regimens should be considered when there is an absolute indication for antiplatelet therapy.
